# The effect of a hiding space on the behaviour and heart rate variability of dairy calves during temporary separation from the dam

**DOI:** 10.1017/awf.2023.100

**Published:** 2024-01-16

**Authors:** Hannah B Spitzer, Rebecca K Meagher, M Lynne O’Sullivan, William Montelpare, Miriam B Gordon, Shawn LB McKenna, Kathryn L Proudfoot

**Affiliations:** 1 Department of Health Management, Atlantic Veterinary College, University of Prince Edward Island, 550 University Ave, Charlottetown, Canada; 2 Department of Companion Animals, Atlantic Veterinary College, University of Prince Edward Island, 550 University Ave, Charlottetown, Prince Edward Island, Canada; 3 Department of Animal Science and Aquaculture, Faculty of Agriculture, Dalhousie University, 62 Cumming Drive, Bible Hill, Nova Scotia, Canada; 4 Health Research Network, Applied Human Sciences, University of Prince Edward Island, 550 University Ave, Charlottetown, Canada

**Keywords:** animal welfare, cow-calf contact, heart rate variability, lying behaviour, parturition, sleep

## Abstract

In natural settings, newborn calves hide for several days before joining the herd. It is unclear whether dairy calves housed indoors would show similar hiding behaviour. This study aimed to describe the use of an artificial hide provided to calves during temporary separation from the dam and assess the effect it has on lying and sleep-like behaviour, as well as heart rate variability (HRV). Twenty-eight cow-calf pairs were randomly assigned to having a hide (n = 14), or no hide (n = 14). Hide use (n = 14), as well as lying and sleep-like behaviour (n = 28), were recorded continuously via video camera during the first hour after the dam was removed for morning milking on day three to seven. Heart rate and R-R intervals were recorded using Polar equine monitors for a subsample of 12 calves (n = 6 per treatment) on day six. Descriptive statistics were calculated for hide use. Wilcoxon Signed Rank tests were used to evaluate whether having a hide affected lying and sleep-like behaviours as well as HRV. Hide use decreased over days and was highly variable between calves. Lying behaviour did not differ between treatments. Duration of sleep-like behaviour was higher for calves without a hide compared to those with a hide. Calves with a hide tended to show signs of higher HRV and parasympathetic activity compared to calves without a hide. Results suggest that providing a hiding space to young calves may be beneficial during periods when the cow is removed from the pen for milking.

## Introduction

In many dairy production systems, calves are typically housed in environments that differ greatly from nature (for a review, see Whalin *et al.* 2021). For example, calves are often separated from their dams within hours of birth; however, public concern for animal welfare (Sirovica *et al.* 2022) has lead researchers to investigate ways for cows and calves to have contact to allow for the expression of natural behaviours (for a review of ‘cow-calf contact’ systems, see Sirovnik *et al.* 2020). A main challenge of cow-calf contact systems is determining how best to house the cow and calf together while still allowing the cow to be milked. For example, some researchers allow full contact between the pair while milking the cow either in her own pen (e.g. Wenker *et al.* 2022) or temporarily separate the cow from her calf to be milked (e.g. Roadknight *et al.* 2022). It remains unclear how these different options might impact the welfare of the calf.

In more natural settings where the calf remains with the cow, such as feral cattle and wild ungulates, calves will either remain hidden or follow the dam in early life, depending on the resources available to them (for a review, see Rørvang *et al.* 2018). For example, in a feral herd of Maremma cattle, calves were often found during the first few days of life to be hidden under bushes for most of the day while the dam grazed close by, but some calves followed the dam by three to four days of life (Vitale *et al.* 1986). For the first five days of life, the calf and dam remained close to each other, but as the calves aged, the dam spent progressively more time grazing with the herd. For the first ten days of life, the calf was regularly > 15 m away from the dam but had the option of natural cover while the dam was away. In a commercial setting, there is also evidence that dairy calves will use an artificial hide in the first few hours of life when kept with their dams (Jensen & Rørvang 2018). However, in cow-calf-contact systems where the dam is separated from the calf to be milked, it is unclear whether a calf would use or benefit from a hiding space while the dam was away for a short milking period.

Some researchers have assessed calves’ responses to temporary separation from their dam in cow-calf contact systems. For example, Roadknight *et al.* (2022) found that calves separated for half a day had a longer latency to lie down after the dam was removed for morning milking compared with calves that were only separated from the cow for milking. The authors speculate that calves separated for half a day were more aroused during separation than those separated only for milking. Lying behaviour may provide a degree of insight into calf arousal; however, changes in lying behaviour are not straightforward indicators of cattle welfare, as lying time can indicate both positive states, such as relation as well as negative states such as pain associated with lameness (Tucker *et al.* 2021).

In addition to lying behaviour, sleep-like behaviours may provide additional information regarding how separation from the cow may affect the calf. For example, there is evidence that calves kept with their dams have longer, less fragmented sleep than those separated in early life (Hänninen *et al.* 2008a), suggesting that changes in sleep-like behavior may enable us to determine when calves are experiencing distress. Measurements of the autonomic nervous system, such as heart rate and heart rate variability (HRV) can also provide insight into how calves respond to their environments and stressors such as disbudding pain (Stewart *et al.* 2009). To our knowledge, as yet there has been no research assessing the effect of a hiding space on lying behaviour, sleep-like behaviour, or HRV during temporary separation from the dam.

The objectives of this study were to: (1) describe how dairy calves use an artificial hide during temporary separation from the dam across the first week of life; (2) assess the effect of a hide on calves’ lying and sleep-like behaviour; and (3) assess the effect of a hide on calves’ heart rate and heart rate variability before and during separation from the dam. We expected calves to use a hide when separated from the dam if provided one, and that use of the hide will decrease throughout the first week of life. We also hypothesised that calves given a hide would experience less distress during separation and would therefore be expected to spend more time lying, have a shorter latency to lie down, spend more time performing sleep-like behaviour as well as have a lower and more variable heart rate compared with those without a hide.

## Materials and methods

### Study animals and housing

This experiment took place at the Dalhousie University Ruminant Animal Centre (Truro, NS, Canada) between September 2021 and September 2022. The animals were cared for in accordance with the guidelines of the Canadian Council of Animal Care (Dalhousie protocol #1031750; University of Prince Edward Island protocol #20-026).

**Figure 1.**
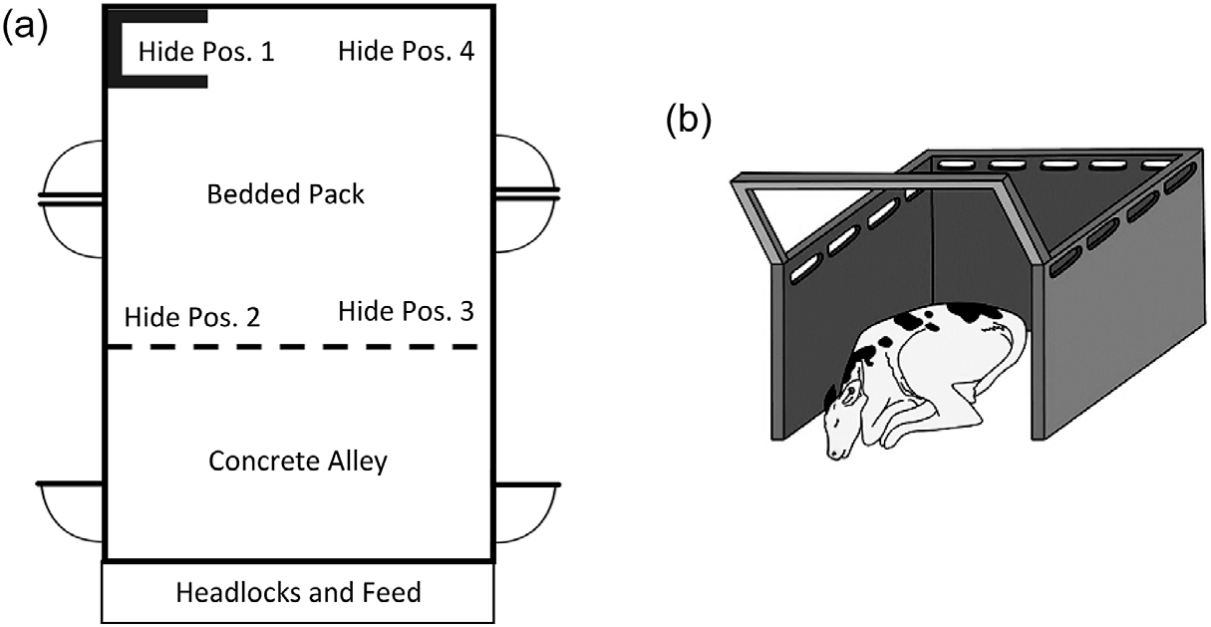
The layout of the experimental pen (a) and the design of the hide (b). For cow-calf pairs in the hide treatment, the hide was placed randomly in one of four possible hide locations (‘Hide Pos’ 1–4). The hide was constructed from three green plastic panels (each 107 × 76 cm [height × width]; MS Schippers, Lacombe, Canada) bolted together in a ‘U’ shape. The quarter-circle shapes on the outside of the pen indicate gates.

A total of 37 calves were enrolled in this study (18 female, 19 male). Calves were either Holstein (n = 18) or Angus-Holstein cross (n = 19). The experimental housing area consisted of one large straw-bedded pack pen (approximately 134 m^2^) which was connected via a gate to an outdoor dry lot (approximately 360 m^2^). Gates could be utilised to divide the bedded pack area into four equally sized smaller pens (34 m^2^). Visual and physical contact with neighbouring cows or cow-calf pairs was possible through the gates. Throughout the experiment, between one and three of these smaller pens (‘experimental pens’; Figure 1[a]) were used to house one cow-calf pair at a time. Each experimental pen consisted of a bedded pack area (22 m^2^), a concrete feed alley (12 m^2^), a feed bunk with four headlocks, and one water dispenser. The back wall of each experimental pen was made of solid concrete and the side walls of the pens were gates covered in plastic mesh to prevent the calves from slipping through or under the gates. Pens were spot-cleaned three times daily and additional straw was added as needed to maintain a dry environment; pens were fully scraped and bedded with new straw twice weekly.

The week prior to their expected calving date, cows were transferred into the large bedded-pack area adjacent to the experimental pens. Cows were then trained once daily to move between the maternity pen and the milking wing to ensure that they were accustomed to this process. Training was carried out by luring cows with a food reward of lactating cow TMR as needed and cows were considered trained when one handler could readily move them between the maternity pen and the milk wing. All cows were outfitted with an udder net to minimise the amount of milk suckled by the calf to align with farm protocol. The udder net was put in place during the week prior to expected calving to allow the cow to become habituated to its presence. Upon signs of calving, cows were moved into one of the three experimental pens (typically into the furthest available experimental pen in order for sufficient space to be left for remaining pre-parturient cows). Seven cows (four in the hide treatment and three in the no hide treatment; treatments described below) calved in the group pen and were moved into the experimental pen within 2 h after calving. Cows and calves were kept together in the same experimental pen until seven days after calving.

On the day of calving, the cow was milked in the experimental pen using a portable milking unit. Each cow was milked within 2 h of calving, and again at 1530h if the cow calved before 1200h. Colostrum was tested using a Brix refractometer (Atago Pal-1, Japan) with a minimum Brix score requirement of 25 before the colostrum was fed to the calf (Quigley *et al.* 2013). All cows met the colostrum quality requirement, and the calf was fed 4 L of the dam’s colostrum within 2 h of birth. We opted not to allow the calf to suckle from the dam and used the udder net to prevent suckling as per the farm’s protocol regarding the ability to control the consistency of milk being fed to each calf. To reduce the likelihood of calves attempting to suckle from the dam, we fed them 2 L of milk or milk replacer with a bottle as often as the farm was able (four times daily) at approximately 0400, 0930, 1530, and 2200h. For the first four days of life, the calf was fed milk from the dam to remain consistent with farm protocol. From day five onwards, the calf was fed milk replacer with a bottle (Milk Chow 26-19, Purina, Mississauga, Canada). In experimental pens, cows were delivered fresh TMR (total mixed ration) four times daily at approximately 0400, 0930, 1200, and 1500h.

Cows were milked twice daily at approximately 0430 and 1600h. The first two milkings were carried out in the experimental pen using a portable milking unit. Subsequently, the cow was removed from the pen to be milked in a tie-stall milking wing. The cow was absent from the pen for approximately 1.5 h per milking (mean [± SD]; 94 [± 27] min; range: 60–168 min). On day seven after giving birth, the cow was moved to the milking wing for the PM milking as usual but stayed there permanently after milking. The calf was then taken from the experimental pen to the calf barn. To help reduce separation distress, where possible the calf was housed with a partner calf.

### Experimental design

Before calving, cow-calf pairs were allocated to one of two treatments: provision of a hiding place for the calf (hide treatment) or no provision of a hiding place for the calf (no hide treatment). Cow-calf pairs were allocated into treatments semi-randomly based on expected calving date, balancing the treatments for breed and sex of calf as best as possible. Information regarding the dam’s parity as well as the calf’s breed and expected sex were provided by the farm staff prior to expected calving. The farm used Angus bull semen in 65% of their cows and female-sexed semen for approximately 17% of the herd.

The hide was constructed from three green plastic hog sorting panels (each 107 × 76 cm [height × width]; MS Schippers, Lacombe, Canada) bolted together in a ‘U’ shape. The hide was attached to the gate of the experimental pen via straps, and a steel bar in an inverted ‘U’ shape was fitted across the entrance to the hide for support and to prevent the cow from entering the hide (Figure 1[b]). Since this was the first research project to our knowledge to assess the use of a hide in calves kept with their dams during the first week of life, the decision was taken to test different positions of the hide within the pen in case this affected the calves’ use of the hide. Thus, the hide was randomly assigned to one of four locations in the experimental pens (in one of the back two corners of the bedded pack area or one of the two front corners of the bedded pack area of the pen, all facing the centre of the pen; Figure 1[a]). We did not have specific predictions as to whether the calves would use the hide more in a certain area but wanted to include a comparison of these locations as preliminary data for future projects assessing hide preference.

### Behavioural observations

Each experimental pen was equipped with two video cameras (Lorex Technology, Markham, Canada) which were positioned approximately 3 m above the pen. One camera was positioned above the bedded pack and captured the top half of the pen while the second was sited above the headlocks and captured the bottom half of the pen. In pens with a hide, a third camera was deployed, positioned directly above the hide to record the interior. Video data were analysed using the ethogram described in Table 1. Behavioural data, including hide use, lying, and sleep-like behaviour, were collected for 60 min continuously for each calf starting when the cow was removed from the pen for morning milking (0430h) on days three to six of life. Data were not collected on days one or two because the dam may have still been milked in the experimental pen on those days depending on the time of day that they gave birth (e.g. for cows that calved later in the day their second milking would have been on day two). Behavioural data were only collected during morning milking to reduce the chance of there being activity in the barn and to avoid time-periods when the calf was wearing the heart rate monitor. Behavioural data were collected by two trained observers. Inter-observer agreement was assessed for all behaviours using an initial sample of nine calves on day three (three no hide calves and six hide calves); the inter-observer correlation was *r* = 0.96, suggesting good agreement between raters’ estimates of calf behaviours.

**Table 1.** Ethogram for behavioural observations of the calf while the dam is removed for milking. Behaviours in the category ‘hide use’ were scored only for the calves in the hide treatment

Category	Position	Description
Hide use^1^	In hide	Fully inside hide: 50% or more of calf’s body is inside the hide Partially inside hide: Part of, but less than 50%, the calf’s body is inside the hide
	Near hide	The calf is within one calf length of any side of the hide (including the open side); the calf must become stationary during the bout for a bout to be included
Sleep-like behaviour^2^		Resting with head lifted and still, or resting with neck relaxed (head resting on ground or tucked against body; disregarding ear and eye movement)
Lying^3^		Lying on sternum or side, head may be rested or raised (when calf is transitioning from lying to standing, the calf is considered lying until supported by all four legs)

1 Modified from Vinke *et al.* (2014)

2 Modified from Hänninen *et al.* (2008b)

3 Modified from Wenker *et al.* (2021)

### Heart rate and heart rate variability

Heart rate (HR) and heart rate variability (HRV) were collected from calves on days three and six after birth during afternoon milking (1600h) using Polar H7 heart rate monitors fitted onto a Polar equine belt. However, data collected from day three were unusable due to technical challenges associated with the equipment and not included in the analysis. Data from the monitors were collected and stored remotely using a Polar Unite watch (Polar, Helsinki, Finland). Prior to placing the belt on the calf, lubricant (Muko, Toronto, Canada) was applied liberally to both the belt and the calf to optimise conductivity. The belt was fitted onto the calf by looping twice around the heart girth, ensuring that the electrode surface remained in contact with the calf. The monitor was placed on the calf approximately 2 h before the cow was removed from the pen for milking (e.g. at 1400h) to allow the calves a 1-h period to become accustomed to the equipment (see Kovács *et al.* 2014) as well as to allow the collection of a 1-h ‘baseline’ period prior to removing the cow from the pen. The belt remained on the calf for 1 h after the cow was separated (‘separation’ period).

Heart rate data were uploaded and stored on the Polar Flow website and an external hard drive. Kubios software was used to analyse the data (Kubios HRV Standard version 3.5). The Kubios software beat correction function was used to identify and correct artifacts in the data; the beat correction threshold was set to ‘very low’ to minimise removal of natural variation (Jimenez *et al.* 2019); the software also corrected the data using a cubic spline interpolation. To estimate HRV, we used time-domain, frequency-domain and non-linear component analysis which have previously been reported in dairy cattle (for a description, see Kovács *et al.* 2014). Time-domain measures included heart rate (HR; bpm), the R-R interval (intervals between successive heartbeats or RR; ms), and the root mean square of successive differences (RMSSD; ms). Frequency-domain measures included the ratio (LF:HF) of low- (LF, 0.04–0.29 Hz) and high-frequency power (HF, 0.30–0.80 Hz). The low and high frequency power ranges were selected based on previous studies in calves (Kovács *et al.* 2014). Non-linear component analysis included the standard deviation of Poincaré plot perpendicular to the line-of-identity (SD1) and the standard deviation of the Poincaré plot along the line-of-identity (SD2) as well as the SD2/SD1 ratio. In addition to these measures previously reported in cattle, we also used the Kubios software to generate parasympathetic (PNS) and sympathetic nervous system (SNS) indexes. The PNS index is calculated using the mean RR, RMSSD, and SD1; the SNS index is calculated using the mean HR, Baevsky’s stress index, and SD2. These indexes are yet to be validated in cattle but have been for use in humans where they are associated with acute stress measures (e.g. Ayuso-Moreno *et al.* 2020). Thus, we decided to include them in this study as they could provide insight into complex arousal states that may be overlooked with single variables, but we do note that these results should be interpreted with caution.

Heart rate data were analysed in 5-min time windows, with a maximum average error rate of 5% for each window (i.e. the data do not contain more than 5% anomalies), as recommended by von Borell *et al.* (2007). Two 5-min windows were selected for each calf, one in the baseline period and one in the temporary separation period when the dam was removed from the pen for afternoon milking on day six. In the baseline period, we used the first 5-min period in which the calf was lying down (not showing sleep-like behaviour) to minimise artifacts (von Borell *et al.* 2007). For the separation period, we used the 5-min window starting 5 min after the dam was separated for milking, regardless of the position of the calf; this enabled us to record a consistent time-period for each calf relative to the cow being removed from the pen.

### Exclusion criteria and matched samples

Cow-calf pairs were excluded from the study if either the cow or calf displayed signs of clinical disease (n = 3). Calves were assessed for health once daily using the Wisconsin Dairy Calf Health Scoring chart (University of Wisconsin-Madison, Madison, WI, USA) by one trained observer. Calves were to be excluded if they required treatment based on the scoring chart guidelines (total respiratory score of five or more, or a faecal score of three); however, no calves met this criterion. Cows were examined by the farm veterinarian if demonstrating signs of poor health (e.g. inability to stand or high body temperature); three cows (two no hide treatment, one hide treatment) were diagnosed with acute hypocalcaemia by the farm veterinarian after calving resulting in both the cow and calf being removed from the study. Cow-calf pairs were also excluded for safety reasons if the dam became too agitated to be separated from milking (n = 1, hide treatment), there was too much human disturbance for the calf while the cow was separated for milking (n = 1, hide treatment), or if more than 50% of the video data were missing due to technical issues (n = 3; two hide treatment, one no hide treatment).

For the remaining 29 cow-calf pairs, matched samples were created by first matching calves in each treatment for sex (male and female) and then breed (Holstein or Holstein-Angus cross). This resulted in the exclusion of one cow-calf pair from the hide treatment for not having a match; this pair was randomly selected from the possible pairs to exclude. This matching resulted in each treatment containing 14 calves (eight females and six males per treatment, as well as seven Holstein and seven Angus-Holstein calves per treatment) from 14 dams (hide: six primiparous, eight multiparous, no hide: seven primiparous, seven multiparous). Data from these 28 cow-calf pairs were used for the hide use and behavioural data analysis.

Further exclusions were made to the 28 cow-calf pairs for the heart rate data due to technical difficulties with using the equipment. Calves were excluded from the heart rate data analysis when the Polar H7 HR monitor did not connect to the Polar Unite watch (n = 3; one hide treatment, two no hide treatment) or because the monitor dropped connectivity potentially as a result of displacement of the belt (n = 12; seven hide treatment, five no hide treatment). Of the remaining 13 calves, there were six and seven in the no hide and hide treatments with HRV, respectively. The six calves in the no hide treatment with HRV data, along with their matched pair from the hide treatment, were used as a subsample of calves to assess heart rate and heart rate variability data on day six after birth (n = 6 matched samples per treatment). Five of these six pairings were matched both for sex and breed (one Holstein-Angus heifer, one Holstein heifer, two Holstein-Angus bulls, and one Holstein bull in each treatment), and the last pairing was matched for sex (bull) but not breed (the calf in the hide treatment was a Holstein-Angus and the calf in the no hide treatment was a Holstein).

### Data analysis

All statistical analyses were performed in SAS Online for Academics (version 3.6; SAS Institute Inc, Cary, NC, USA), with calf as the experimental unit. Prior to analysis, all data were summarised by calf and day (day three to six for behavioural data and day six for HRV data). Data were then visually assessed for normality using the raw data and residuals. Both the behavioural and HRV data were generally not normally distributed, so descriptive and non-parametric statistics were used and a statistical significance level of *P* < 0.05 was set *a priori.*

Descriptive statistics were used for hide use (Table 1) across days (three to six). Hide use data were categorised into two behaviours: in hide and near hide. Using the PROC MEANS statement in SAS, descriptive data including mean, standard deviation, min, and max were calculated. The number of calves (and percentage) that entered the hide during temporary separation from the dam during milking were calculated manually using Excel® and hide use based on the location of the hide in the pen (using positions 1, 2, 3 or 4 from Figure 1[a]). To determine if the position of the hide influenced the calves’ use of the hide, the difference in hide use between hide positions was determined by producing an area under the curve (AUC) for each calf using the following approach. Due to a low sample size, we decided to collapse the four hide positions into two categories that we were most interested in comparing: front (hide positions 2 and 3) and back (hide positions 1 and 4). Missing observation days (eight observation days out of 112, four hide [two back position, two front position] and four no hide) were filled by taking the average of the three other observation days for the calf. A Wilcoxon Signed Rank test of the AUC (one-tailed) was then used to determine differences in time spent in the hide and time spent near the hide between front and rear hide positions. Results are reported as *Z* scores and probability values.

Next, the area under the response curve (AUC2) was calculated for lying behaviour (lying duration, the number of lying bouts, lying bout duration, and the latency to lie down after the dam was removed from the pen) and for sleep-like behaviour (duration of sleep-like behaviour, the number of sleep-like bouts, and the average duration of sleep-like bouts). A Wilcoxon Signed Rank test of the AUC2 (one-tailed) was then used to determine differences in lying and sleep-like behaviour between treatments. Results are reported as *Z* scores and probability values.

In the third analytical approach, Kubios software was used to produce the following variables for each calf in the baseline and separation periods: mean HR, mean RR, RMSSD, LH:HF, SD2/SD1, PNS index and SNS index. The difference in each of these variables between the baseline and separation was calculated for each calf before analysis (separation–baseline). A Wilcoxon Signed Rank test (one-tailed) was then used to determine the effect of treatment on these variables during the baseline period, the separation period, as well as the change between the two periods. Results are reported as *Z* scores and probability values.

## Results

### Hide use

**Figure 2.**
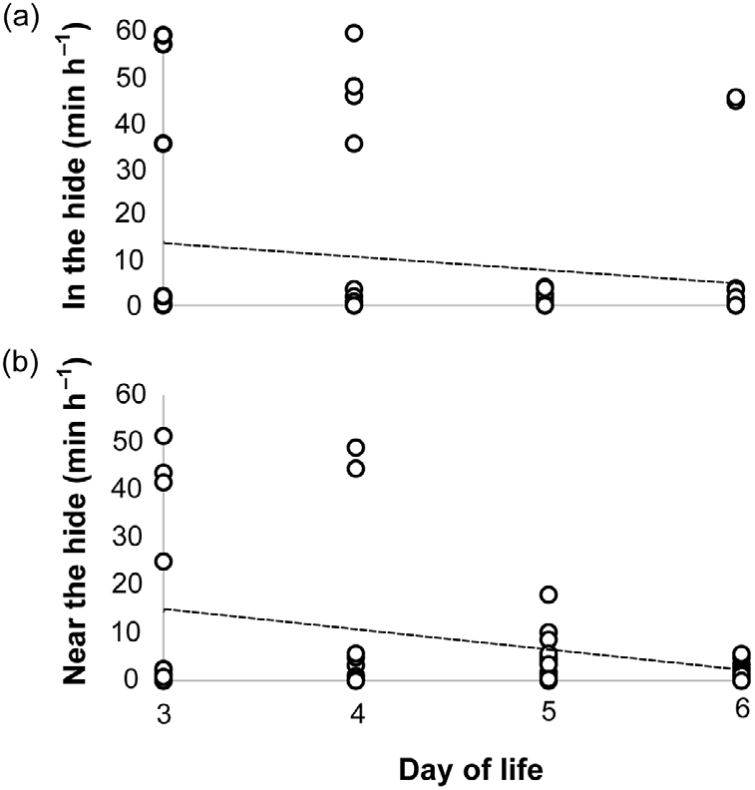
The time that each calf (n = 14 in ‘hide’ treatment) spent (a) inside the hide (at least half the body was inside the hide) and (b) near the hide (calf was within one calf length of the hide) on days three to six of life during the 60-min period of temporary separation from the dam. Circles represent individual calves, and dotted lines represent the trendline for each dataset.

The amount of time that calves spent inside and near the hide on days three to six of life is shown in Figure 2. Hide use decreased over the four-day observation period (in hide slope = –2.91; near hide slope = –4.14). Hide use was highly variable between calves; Table 2 shows the number of calves that performed at least one ‘in hide’ bout and at least one ‘near hide’ bout across days.

**Table 2.** Number (and percentage) of calves in the hide treatment (n = 14) that went inside the hide (at least half the body was inside the hide) or near the hide (calf was within one calf length of the hide) at least once during the 60-min period of temporary separation from the dam from day three to six of life

Day	In hide	Near hide
3	7 (50%)	11 (79%)
4	8 (57%)	11 (79%)
5	7 (50%)	13 (93%)
6	8 (57%)	12 (86%)

Calves with a hide in a back (n = 7) and front (n = 7) position spent similar amounts of time in the hide (*Z* = –0.76; *P* = 0.22) and near the hide (*Z* = 0.51; *P* = 0.30).

### Lying and sleep-like behaviour

**Figure 3.**
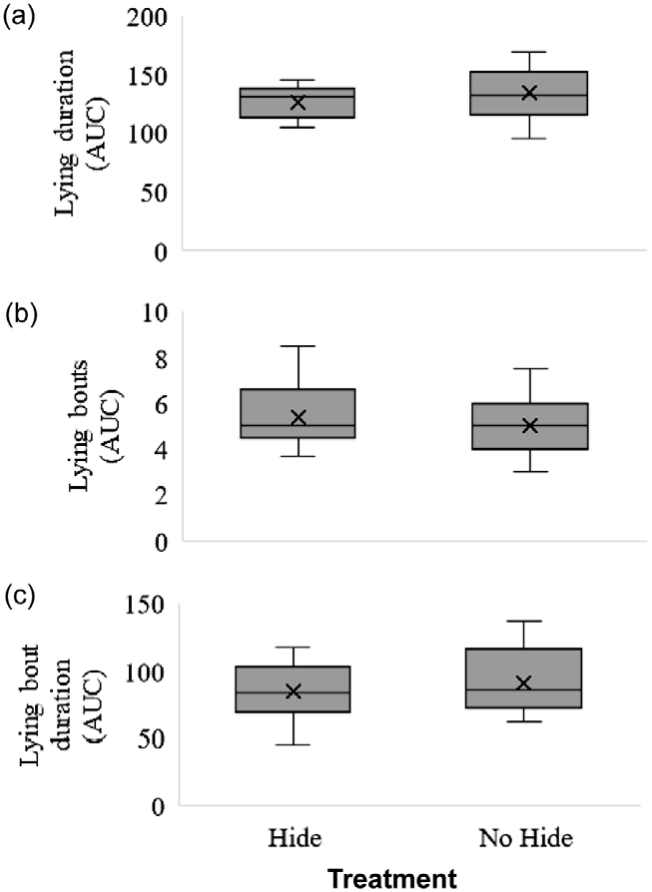
The distribution of the area under the curve (AUC) for (a) the duration of lying, (b) the number of lying bouts and (c) the average duration of lying bouts for dairy calves given a hiding place (hide) or not (no hide) during a 60-min period of temporary separation from the dam on days three to six of life. Upper and lower box limits represent the first and third quartiles. The black line within each box represents the median, and the x represents the mean. Whiskers extend to the lowest and highest values that are not outliers (values that are 1.5× the interquartile limits).

There were no differences in lying behaviour between calves with and without a hide (Figure 3), including the lying duration (*Z* = –0.80; *P* = 0.21), the number of lying bouts (*Z* = 0.46; *P* = 0.32), the lying bout duration (*Z* = –0.53; *P* = 0.30) and the latency to lie down after the dam was removed from the pen (*Z* = –1.04; *P* = 0.15).

**Figure 4.**
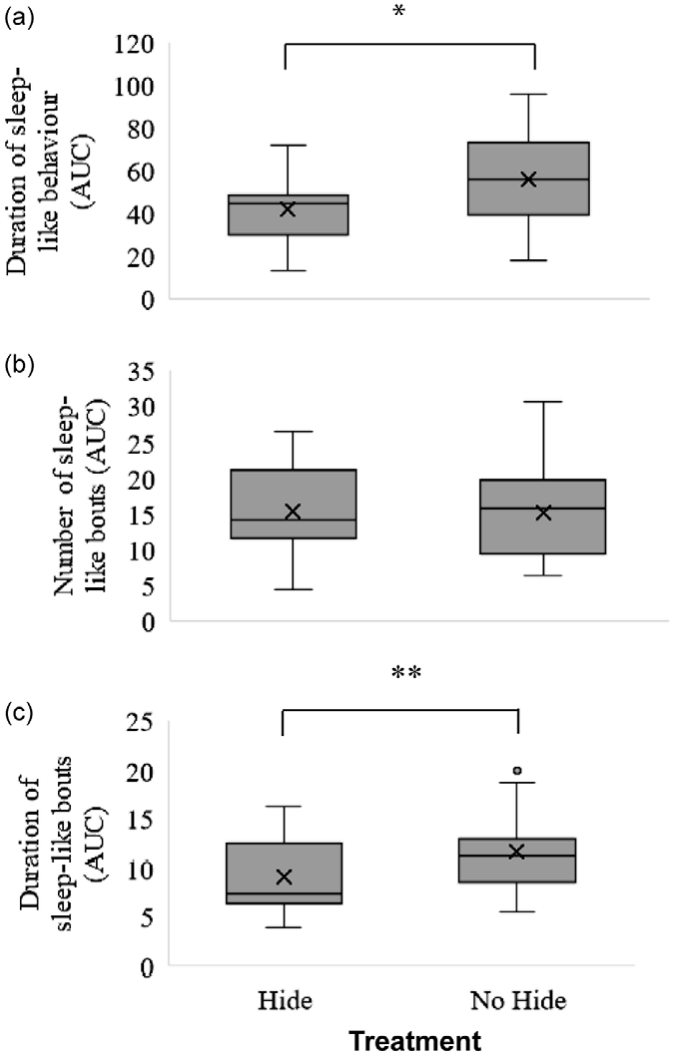
The distribution of the area under the curve (AUC) for (a) the duration of sleep-like behaviours, (b) the number of sleep-like bouts, and (c) the duration of sleep-like bouts for dairy calves given a hiding place (hide) or not (no hide) during the 60-min period of temporary separation from the dam on days three to six of life. Upper and lower box limits represent the first and third quartiles. The black line within each box represents the median, and the x represents the mean. Whiskers extend to the lowest and highest values that are not outliers (values that are 1.5× the interquartile range). Circle outside of the whiskers indicates an outlier. ** Variables that show tendency to differ between treatments (0.05 ≥ *P* ≤ 0.1). * Variables that differ between treatments (*P* < 0.05).

The distribution of AUC2 scores for calves in the hide and no hide treatment for sleep-like behaviours during the 60-min period of temporary separation from the dam is shown in Figure 4. Calves without a hide spent more time performing sleep-like behaviours than those with a hide (*Z* = –1.91; *P* = 0.03). The number of sleep-like bouts did not differ between treatments (*Z* = 0.30; *P* = 0.38), but the duration of sleep-like bouts (*Z* = –1.68; *P* = 0.05) tended to be longer in calves without a hide compared to those with a hide.

### Heart rate and heart rate variability

The mean, standard deviation and *P-*values of the time domain parameters (HR, RR, RMSSD), frequency domain parameters (LF:HF), the non-linear parameters (SD2/SD1), PNS and SNS indexes during the baseline and separation periods are shown in Table 3, as well as the change in these parameters between the two periods, for calves with and without a hide.

**Table 3.** Mean (± SD) heart rate and heart rate variability measurements of dairy calves given a hiding place (hide) or not (no hide) before (baseline; first 5-min lying bout in the hour preceding separation) and during (separation; 5 min after dam removed) the 60-min period of temporary separation from the dam. The change between the separation and baseline periods (separation – baseline) is also indicated

	Treatment		
	Hide	No hide	*P-*value
Baseline period			
HR (bpm)	130.2 (± 15.6)	143.0 (± 13.8)	0.06
R-R Interval (ms)	466.2 (± 52.0)	422.8 (± 38.1)	0.06
RMSSD^1^ (ms)	9.4 (± 3.5)	7.1 (± 5.0)	0.19
LF:HF^2^	17.9 (± 15.0)	18.4 (± 23.7)	0.34
SD2/SD1	2.8 (± 1.6)	3.7 (± 1.9)	0.29
PNS Index	–3.5 (± 0.4)	–3.8 (± 0.4)	0.09
SNS Index	8.4 (± 1.4)	11.24 (± 3.4)	0.06
Separation period			
HR (bpm)	143.7 (± 17.8)	158.0 (± 18.3)	0.11
R-R Interval (ms)	422.8 (± 49.3)	383.8 (± 41.9)	0.11
RMSSD (ms)	9.6 (± 5.9)	5.2 (± 2.6)	0.10
LF:HF	31.6 (± 37.2)	28.8 (± 21.8)	0.34
SD2/SD1	4.2 (± 3.0)	3.2 (± 0.9)	0.47
PNS Index	–3.7 (± 0.3)	–4.2 (± 0.3)	0.02
SNS Index	8.9 (± 1.4)	13.4 (± 4.3)	0.02
Change between baseline and separation periods			
HR (bpm)	13.5 (± 25.5)	17.9 (± 1.1)	0.40
R-R Interval (ms)	–43.3 (± 74.0)	–39.0 (± 40.1)	0.40
RMSSD (ms)	0.2 (± 8.1)	–2.0 (± 6.6)	0.50
LF:HF	13.7 (± 38.9)	10.4 (± 32.9)	0.34
SD2/SD1	1.3 (± 3.1)	–0.5 (± 2.5)	0.09
PNS Index	–0.2 (± 0.6)	–0.4 (± 0.2)	0.50
SNS Index	0.5 (± 2.1)	2.2 (± 2.8)	0.24

1 RMSSD = Root mean squared of successive differences in the R-R interval

2 LF:HF = low frequency (0.04–0.29 Hz) to high frequency (0.30–0.80 Hz) ratio

Baseline HR tended to be higher for calves without a hide compared to those with one (*Z* = –1.52), yet no differences in HR were seen either during the separation period (*Z* = –1.2) or in changes in HR between baseline and separation periods (*Z* = 0.24).

There were no differences between treatments for baseline RMSSD (*Z* = 0.89), yet calves with a hide tended to have higher RMSSD during the separation period compared with those without a hide (*Z* = 1.28). There were no differences between treatments in the change in RMSSD between the baseline and separation period (*Z* = 0.00).

Calves with a hide tended to have a higher baseline RR (*Z* = 1.52) compared to those without a hide. There were no differences between treatments for separation RR (*Z* = 1.20) nor in the change in RR between the baseline and separation periods (*Z* = –0.24).

There were no differences between treatments for baseline LF:HF (*Z* = 0.40), separation LF:HF (*Z* = –0.40), or the change in LF:HF (*Z* = –0.40) between the baseline and separation periods.

There were no differences between treatments for baseline SD2/SD1 (*Z* = –0.56) or separation SD2/SD1 (*Z* = –0.08); however, the change in SD2/SD1 tended to be higher for calves with a hide compared with those without a hide (*Z* = 1.36).

Calves without a hide tended to have a higher baseline SNS index (*Z* = –1.52) and had a higher separation SNS index (*Z* = –2.48) compared to those with a hide. There were no differences between treatments for the change in SNS index between baseline and separation periods (*Z* = –0.72). In contrast, calves with a hide tended to have a higher baseline PNS index (*Z* = 1.36) and had a higher separation PNS index (*Z* = 2.16) compared to those without a hide. There were no differences between treatments for the change in PNS index between baseline and separation periods (*Z* = 0.00).

## Discussion

The objectives of this study were to describe how indoor-housed, dam-reared dairy calves use an artificial hide, as well as to determine the effect of the hide on lying, sleep-like behaviours, and heart rate variability parameters during temporary separation from the dam during the first week of life. Use of a hide was variable between calves and tended to decrease over the first week of life. The presence of a hide did not affect lying behaviour of calves when their dams were removed for milking, but calves without a hide had longer durations of sleep-like behaviour than calves with a hide. Calves with a hide tended to show signs of lower sympathetic activity and higher parasympathetic activity compared to calves without.

Many, but not all, calves in this study spent time inside and near the hide during temporary separation from the dam, and hide use decreased over the four-day observation period. This hiding behaviour aligns with the natural behaviour of ungulates in the first week of life. For example, research in feral cattle (Vitale *et al.* 1986) and extensively housed deer (Wass *et al.* 2004) has suggested that young ungulates show an inclination to hide for the first several days to a week after birth, but not all calves will hide after birth (see Rørvang *et al.* 2018). After this age, calves likely become more active and are less likely to spend their time hiding. It is unclear from our study if calves or their dams would attempt to join a herd during the first week of life, as the pairs were housed separately from other animals.

Hide use was variable between calves in the study. Jensen and Rørvang (2018) reported that 62% of indoor-housed dairy calves moved to a large artificial hiding area (3 × 4.5 m; length × width) within 3 h after birth. Similarly, Zobel *et al.* (2020) reported that of the 82% of cows that did not calve in a large hiding area (2.7 × 3 m), 73% of cow-calf pairs moved into the hiding area during the day of calving. These two studies reported the number of calves or cow-calf pairs that moved into a hiding area large enough for both the cow and the calf, but the amount of time spent hiding or the variation between calves was not reported. Gingerich *et al.* (2020) found that group-housed calves who were provided with a small hiding area (1.2 × 1.2 m) also demonstrated large individual variability in hide use (10.8 min to 20.7 h per day), although hide use in this study was associated with post-disbudding pain rather than neonatal hiding behaviour. Given the findings of our study and others, it appears that many, but not all, pre-weaned dairy calves will use a hiding space if provided with one. More research could be helpful to understand factors that contribute to hide use in indoor-housed dairy calves during temporary separation from the dam, such as hide size and design, social dynamics, as well as individual differences in calf personality. The position of the hide within the pen may be less important as evidenced by our findings that hide use was not affected by the hide being at the front or the back of the pen; however, future research could assess the use of a hide in the middle of the pen as there is some evidence in deer calves that position of a hide can affect hide use on pasture (Hodgetts *et al.* 2002).

Calves with and without a hide in our study showed similar lying durations, number of lying bouts, duration of lying bouts, and latency to lie down after the dam was removed from the pen for milking. Roadknight *et al.* (2022) found that calves separated from their dams for half a day had a longer latency to lie down after the dam was removed for morning milking compared with calves that were only separated from the cow for milking. Authors interpreted longer latency to lie down as an indicator of distress and arousal in the calves separated for half a day. In our case, calves in both treatments were separated for the same amount of time, indicating that the presence of a hide did not affect their lying behaviour. However, changes in lying behaviour have been considered as both positive and negative indicators of welfare for cattle depending on the circumstances, making it a challenge to interpret (Tucker *et al.* 2021). Thus, although calves in our present study did not demonstrate differences in lying behaviour based on access to a hiding space, it is difficult to conclude that the calves in the two treatments had similar experiences based on this finding.

Contrary to our predictions, calves provided with a hide spent less time performing behavioural indicators of sleep and tended to have shorter sleep-like bouts compared to those without a hide. Assessing sleep in cattle is still understudied, but research has found that sleep and sleep-like behaviours can be influenced by the calves’ environment. For example, Hänninen *et al.* (2008a) found that calves kept with their dams had less fragmented sleep compared with those housed individually. In the same study, these authors also found that calves who were able to suckle colostrum from a nipple bucket showed increased sleep behaviours compared to those fed with a bucket. The authors argued that calves with more positive welfare and greater ability to perform natural behaviours (such as interacting with the dam and drinking milk from a nipple instead of a bucket) have more, and better quality, sleep. Based on this interpretation, calves without a hide may be considered to have improved experiences during separation compared with those with a hide. However, much like lying behaviour, there is also evidence that increased sleep-like behaviour in animals can reflect poor housing conditions and may not necessarily reflect improved welfare through decreased arousal. For example, Visser *et al.* (2008) found that young horses who were stabled for the first time individually spent more time sleeping and standing vigilant compared to pair-housed horses, who spent more time eating.

Although it remains unclear why the calves without a hide in our study showed longer durations of sleep-like behaviour, we speculate that perhaps performing these behaviours (i.e. lying down still with or without head curled) may be an anti-predator strategy for calves that do not have a place to retreat to when their dam is out of sight. As we cannot say for sure that calves performing sleep-like behaviours were engaged in sleep states (e.g. REM or NREM; Hänninen *et al.* 2008b), this behaviour may be a novel indicator of distress in newborn calves. For example, cats will feign sleep when experiencing stress, potentially as a means of monitoring their environment (for a discussion of this, see Horwitz & Rodan 2018). An alternative hypothesis is that the hides may have interfered with the calves’ ability to sleep, as they may have preferred to sleep in a location where they could see other animals. More research is needed to support these ideas in young dairy calves.

There was an indication that sympathetic nervous system activity was higher for calves without a hide (e.g. higher baseline HR, a tendency for higher baseline SNS index, and a higher separation SNS index), and parasympathetic activity was higher for those with a hide (e.g. a tendency for higher baseline R-R interval, separation RMSSD, change in SD2/SD1 and baseline PNS index, as well as a higher separation PNS index). These differences and tendencies indicate that those calves provided with a hide may be showing signs of higher parasympathetic activity, indicative of reduced arousal and possibly reduced stress, compared to those without a hide. Previous research has demonstrated that acute stressors such as disbudding (Clapp *et al.* 2015; Stewart *et al.* 2008), castration (Bergamasco *et al.* 2021), removal of a dummy teat and reintroduction to conspecifics after isolation (Clapp *et al.* 2015) have resulted in increased indicators of sympathetic activity in calves. Slightly higher sympathetic activity in the calves without a hide may indicate a generally higher state of arousal both before and after the dam was separated. These findings support the idea that calves without a hide may be showing signs of increased vigilance both before and after the dam was removed from the pen.

There are several limitations to this study. Firstly, our ability to interpret the results was affected by the low sample size, especially for the heart rate data. Stewart *et al.* (2008) detected differences in calf HRV between treatments using similar sample sizes (n = 6 to 8 per treatment), however, that particular study was investigating the effect of disbudding on calves which is likely a severe acute stressor that may have a larger effect on heart rate variables compared with temporary separation from the dam. In contrast, Adcock and Tucker (2022) reported no difference in HRV while investigating the effect of a lidocaine injection using a sample size of n = 9 per treatment. With our limited sample size, it is difficult to determine if the detected tendencies indicate real differences between treatments or not. A second limitation of the study was the method used to house the cows and calves. As cattle are social animals, the calves’ behaviour may have been influenced by the lack of other social companions aside from the dam during the first week of life. Sociability develops in calves and increases throughout the first several weeks of life, as calves spend increasing amounts of time with other calves as they age (Vitale *et al.* 1986). Thus, calves’ use of hides and other behaviours may have differed had there been other calves and cows in the same pen. In addition, the number of cows and calves in adjacent pens varied for each calf (e.g. some had another cow-calf pair on one or both sides of the pen while others may have had cows in the dry cow pen next to them on one side), which also may have affected their behaviour.

### Animal welfare implications

There is evidence that the practice of separating the dairy calf from the dam is viewed poorly by the public, suggesting that alternative practices need to be developed that allow for dam-calf contact in early life. As these cow-calf contact systems develop, it is important that we begin to recognise and mitigate stressors that may occur for the dam and their young calves. For many cow-calf contact systems, the dam must be removed during the day to be milked; yet there has been little research into whether this temporary separation negatively affects calves. We believe this to be the first study assessing the effect of a hiding space on indicators of calf stress and arousal in the first week of life. In general, our results suggest that although there was high variability in calves’ use of a hiding space, they seemed to benefit in terms of higher indications of parasympathetic activity when the dam was removed compared with those not provided a hide. Allowing for this natural hiding behaviour may enable calves to cope better when temporarily separated from their dams in cow-calf contact systems. A main limitation of this study was the method used to separate the cow and calf at the end of the study; due to normal farm practices, the pair were separated using an abrupt rather than gradual approach which may have increased distress associated with separation.

## Conclusion

When afforded the opportunity, variable numbers of dairy calves use a secluded place to hide when temporarily separated from the dam, with hiding behaviour shown to vary between individual calves. Similar to the natural environment, hiding behaviour of newborn calves decreases over the first week of life. Calves not provided with a hide spent more time performing behavioural indicators of sleep when the dam was away and tended to have longer bouts of sleep-like behaviour. Calves who were provided a hide had or tended to have higher indicators of parasympathetic activity compared with those calves not provided with a hide. The results from this study indicate that some calves may benefit from having a place to hide when temporarily separated from their dams.
